# No such thing as mental illness? Critical reflections on the major ideas and legacy of Thomas Szasz

**DOI:** 10.1192/pb.bp.115.053249

**Published:** 2016-12

**Authors:** Tony B. Benning

**Affiliations:** 1 Maple Ridge Mental Health Center, Maple Ridge, British Columbia, Canada

## Abstract

Enfant terrible of psychiatry and widely known as one of its most indefatigable as well as iconoclastic critics, Thomas Szasz (1961–2012) had a prolific writing career that extended some 51 years beyond the publication of his first book, *The Myth of Mental Illness*, in 1961. This editorial identifies and critically discusses three major themes in Szasz's writings: his contention that there is no such thing as mental illness, his contention that individual responsibility is never compromised in those suffering from what is generally considered as mental illness, and his perennial interest in calling attention to the political nature of psychiatric diagnosis.

## The non-existence of mental illness

Arguing in *The Myth of Mental Illness: Foundations of a Theory of Personal Conduct* that they are merely ‘indirect forms of communication’,^1^ Thomas Szasz posited that so-called mental illnesses cannot legitimately be categorised as diseases. This launched an argument that Szasz would elaborate over the course of a prolific writing career that spanned more than 50 years. Szasz repudiated psychiatry's misappropriation of concepts such as ‘illness’, which he took to be relevant to medicine and its ‘physicalist framework’^2^ but not to matters of mind and human conduct. In *The Myth of Mental Illness*,^1^ after arguing that virtually any entity can have a counterfeit version, Szasz articulated his views with characteristic iconoclasm, contending that only physical illnesses are real and that mental diseases are ‘counterfeit and metaphorical illnesses’ (p. 34). Illnesses are understood, according to Szasz,^3^ with respect to deviation from a norm, and in the case of physical illness the norm refers to the structural or functional integrity of the body or some aspect of it. But the norm – deviation from which results in so-called mental illness – is altogether more problematic for Szasz; this norm is a ‘psychosocial and ethical one’.^3^ With this as the case, first, the search for a medical remedy seems poorly justified, and second, the points where diagnostic lines are drawn are bound, according to Szasz, to be somewhat arbitrary.

Szasz did not deny that humans have difficulties but he preferred to conceptualise them not as mental illnesses or as diseases, but as ‘problems in living’.^1^ Nor did he deny psychiatrists a role in assisting individuals with problems. Psychiatrists could have a legitimate role to play but the ideal relationship between psychiatrist and patient, for Szasz, should be based on consensual contract rather than coercion. Second, the psychiatrist cannot justifiably claim that only he has the expertise to help people experiencing problems in living, since help from ‘family members, friends, clergymen, mental health professionals, physicians, drugs, religion, faith healing, marriage, divorce, and so on’^4^ could be legitimately solicited by or on behalf of those experiencing problems in living, according to Szasz.

As Kendell^5^ pointed out in his rebuttal of Szasz's claim that mental illnesses fail to conform to the definition of disease, Szasz's notion of disease, influenced by Virchow, was overly narrow, for it placed excessive importance on the criterion of ‘cellular pathology’.^5^ Kendell also claimed that Szasz's argument understated the extent to which ‘suffering and incapacity are fundamental attributes of disease’.^5^ He brought attention to the fact that in medicine generally there is no universally agreed upon definition of disease – and if Szasz's criterion were to be widely adopted, several diseases widely recognised in medicine, such as migraine and torticollis (neither of which are associated with lesions or cellular pathology) would fail to qualify.

Beginning in the 1990s, psychiatry saw an unprecedented shift in its culture, with an explosion of interest in biological and neuroscientific research. Shorter,^6^ on the basis of the claim that ‘the discipline today acknowledges a neurological basis for much psychiatric illness’ (p. 183), argued that contemporary neurobiological research findings in fact provide support to counter Szasz's argument. This view that Shorter attributed to ‘the discipline’ is arguably overstated, since there is by no means unanimity among psychiatrists in attributing biological aetiologies to mental illnesses. For example, several authors writing as recently as early 2000s have contested the very claim that schizophrenia is a brain disease.^7,8^

## Pathologisation of everyday life

A further area examined by Szasz concerns the proliferation of new psychiatric disorders. Szasz decried ‘fictitious mental diseases’^9^ such as body dysmorphic disorder, multiple personality disorder and frotteurism. What he appeared to be objecting to here is the encroachment of psychiatric discourse upon ever-increasing domains of human life. Szasz adopted a mocking tone, for example, when discussing ‘the behavior we call shoplifting – but psychiatrists call kleptomania’,^9^ arguing that the construal of such behaviours as pathological entities for one thing perpetuates an assumption that the individual's actions are devoid of motivation and that they occur outside the orbit of their control. Medicalisation hands responsibility for such behaviours away from the individual concerned to physicians, specifically to psychiatrists.

In this respect, Szasz was cognizant of the interplay between diagnosis and political and social power; medicalisation gives a pre-eminent role to doctors, it privileges the role of medication as a therapeutic intervention and so the pharmaceutical industry stands to profit much by stretching the boundaries of the concept of mental disorder. Several authors in addition to Szasz have brought critical attention to this ever-widening reach of psychiatric diagnosis, and to the pharmaceutical industry's complicity in this phenomenon,^10–14^ not only by supporting ‘new’ categories of psychiatric disorder (such as adult attention deficit disorder), but by endorsing the lowering of diagnostic thresholds for a host of established psychiatric disorders, such as bipolar disorder.

## Mental illness and personal responsibility

Ever interested in the interface between psychiatry and the law, another recurring theme in Szasz's writing concerns the issue of individual agency and personal responsibility. *Law, Liberty, and Psychiatry*^3^ is his first book-length attempt to grapple with these important issues. In it, Szasz articulated a critical position with respect to legal and jurisprudential orthodoxy which assumes that individuals with mental illness, in some cases, are not responsible for their actions (especially with reference to criminal acts). Distinguishing between explanation and responsibility, Szasz argued that regardless of a diagnosis of mental illness, individuals are ‘always responsible for their conduct’.^3^

In discussing the hypothetical example of a deluded individual with schizophrenia who shoots several visitors outside the White House acting under the belief that he was pre-emptively shooting communist assassins, Szasz argued that the endorsement of a range of ‘explanations’ does not imply that the person committing the crime did not have responsibility for the act. Critical of what he dubbed the ‘progressive psychiatrization of the law’,^3^ Szasz challenged the sorts of assumptions about personal responsibility in mentally disordered individuals that are reflected in such jurisprudential principles as the M'Naghten and Durham rules. There are other, more wide-reaching, implications of this assumption for Szasz – widespread in his view among psychiatrists and in jurisprudence – about the impairment of personal responsibility in the ‘so-called mentally ill’.^3^ These he articulated in works such as *The Therapeutic State*^15^ and *The Manufacture of Madness*,^16^ arguing that it is on the basis of the false assumption that the so-called mentally ill person ‘is presumed to be incompetent’^15^ to make decisions about treatment that such paternalistic interventions as involuntary hospitalisation and coercive treatments are justified. These concerns gave rise to such pragmatic developments as his concept of the psychiatric will^15^ in which it was proposed that an individual, when well, could document an objection to receiving coercive treatments in the event of falling sick.

## Discussion

Szasz consistently debunked the existence of mental illness on the basis of his assumption of the relevance to mental illness of the criteria of physiological abnormality, or the so-called ‘medical-pathological definition of disease’.^17^ But this line of argument is problematic, with Kendell pointing out that this criterion is of dubious merit in physical disorder, let alone mental disorder. The scope of Szasz's erudition was broad but it did not encompass non-Western conceptualisations of mental disorder. Had it done so, the somewhat idiosyncratic nature of his argument would perhaps have become apparent to Szasz himself, for the criterion of physiological abnormality as a means of validating the existence of mental disorder is anathema to non-Western conceptualisations of mental illness.^18–22^ As such, Szasz could appeal neither to history nor to insights from cultural psychiatry or anthropology as a way of giving credibility to his argument.

Szasz's concerns about the medicalisation of emotional, behavioural and interpersonal problems remain relevant and psychiatrists' engagement with them would be warranted. Vigorous effort continues to be expended in shoehorning a range of human problems into the medical model and resulting over-diagnosis furthers the professional interests of psychiatrists and the pharmaceutical industry. Many contemporary writers^10,12,13^ share a concern for the societal impact of ever-greater number of psychiatric diagnoses and lowering of diagnostic thresholds for existing ones. However, there is a reality and suffering attached to mental illness, to psychological dysfunction, that Szasz's writings simply fail to acknowledge. In this respect, I fully agree with Lieberman: ‘I think Szasz trivializes devastating malfunction – serious mental illness – by dismissing such patients as attention seekers, imposters, and so forth’.^23^ Admittedly, the language of histopathological lesions, of cellular pathology and infectious organisms has very little or no relevance to most psychiatric conditions, but to insist that problems with thought, emotions, drive, impulse control, cognition, perception, behaviour and so forth should be exempted from the rubric of illness, and the assertion that there is no such thing at all as psychopathology, seem entirely unjustified. Szasz might have attracted less opprobrium from psychiatrists and others had he acknowledged the reality of psychopathology and of mental illness while conceding that it does indeed have characteristics that set it apart from many – although not all – physical illnesses. These characteristics include phenomenological overlap with spiritual experience,^24,25^ diagnostic boundaries that are often much less well defined than in physical illness,^12^ boundaries that are subject to the influence of such factors as social context, and so forth. Such characteristics behove clinicians to conceptualise mental illness in different ways from physical illness, not to discard altogether the very concept.

Effort needs to be poured into formulating a conceptual definition of mental disorder^12^ that does justice to serious mental illness so that such *bona fide* psychiatric illnesses as schizophrenia and major depression and mere problems in living are not conflated with each other. This would be a far more humane path along which to proceed than following the highly flawed Szaszian strategy of jettisoning the concept of mental illness.

Psychiatric labels have undoubtedly been deployed in the service of a range of political agendas and Szasz was justified in bringing attention to such historical constructs as ‘drapetomania’ and ‘sluggish schizophrenia’,^17^ but his contention that contemporary psychiatric diagnoses represent nothing more than the labelling of those who are deviating from ‘psychosocial, ethical, or legal norms’^17^ was overstated. This is not a description that resonates with me as a clinical psychiatrist and I doubt that I am alone in this regard. Psychiatrists, contrary to what Szasz appeared to suggest, do not make judgements about pathology on the basis of arbitrary, socially constructed notions of normalcy, conformity and deviance. This is not to say though that there is no concept of a normative reference point. There is, but it usually is assumed as being an approximation to the patient's previous (so-called premorbid) state of health or level of functioning, deviation from which has resulted in distress and/or concern from the patient or their significant others. This is quite a different scenario from the one conceived by Szasz which would appear to portray psychiatrists as playing some sort of role, as agents of social control or as ‘social engineers’.^3^

On the issue of Szasz's views on personal responsibility and on his wholesale dismissal of the claim that individuals diagnosed with mental disorder could ever lack competence to make treatment decisions, I agree with Lieberman^23^ that while the default assumption on the part of physicians should be that patients retain competence, in some circumstances this assumption is proven to be false. In cases when a mentally disordered individual lacks decision-making competency psychiatric intervention may prevent them from harming themselves or others.

The specific question of Szasz's legacy became all the more salient following his death in 2012. Williams & Caplan,^26^ writing in *The Lancet*, credited Szasz with the increasing preoccupation among psychiatrists – especially in the latter part of the 20th century – with disease nosology and with the quest to elucidate biological substrates for mental illness. The justifiability of such attribution of credit in this respect and to this extent to Szasz is debatable since the pursuit of a putative biological basis for mental illness is likely to be explained, as Walter^27^ points out, by several converging streams of influence, recent as well as remote. They include the localationist imperatives that were advanced by Wernicke and Meynert at the end of the 19th century, the psycho-pharmacological breakthroughs of the mid-20th century, and the genetic studies of schizophrenia that soon followed. Williams & Caplan's argument, then, fails to convince. The more persuasive claim regarding his legacy is that which credits Szasz with engendering greater awareness around issues of psychiatric power.^28^ Today, there is growing disquiet about the fact that ‘psychiatric talk’ has come to be a sort of go-to discourse for an ever-widening range of human concerns.^10,12,13^ Undergirding that disquiet is cognizance of the fact that psychiatric power, as Breeding suggests,^28^ need not be overt to be pervasive. As a body of writings that can sensitise us to these important issues, I can think of none more compelling than Szasz's.
